# Supplementary material for: Emotional connectedness and adolescent help-seeking behavior for mental health problems: A situational analysis
Source: PLOS Ment Health. 2026 May 20;3(5):e0000624. doi: 10.1371/journal.pmen.0000624 (PMC13189287; doi:10.1371/journal.pmen.0000624)
Supplement: Attachment — Submitted filename: Response letter.docx [file pmen.0000624.s002.docx]

**Response Letter – PLOS Mental Health
 
 Before we proceed with your manuscript, please address the following prompts:
 
 a) If there are ethical or legal restrictions on sharing a de-identified data set, please explain them in detail (e.g., data contain potentially identifying or sensitive patient information, data are owned by a third-party organization, etc.) and who has imposed them (e.g., a Research Ethics Committee or Institutional Review Board, etc.). Please also provide contact information for a data access committee, ethics committee, or other institutional body to which data requests may be sent.**

**Response: The ethical approval received from the Swedish Ethical Review Agency allows us to share de-identified transcriptions of the focus group discussions in the Swedish language, upon reasonable request. Requests may be sought by contacting** [**dataskydd@hh.se**](mailto:dataskydd@hh.se) **at Halmstad University.
 
 b) If there are no restrictions, please upload the minimal anonymized data set necessary to replicate your study findings to a stable, public repository and provide us with the relevant URLs, DOIs, or accession numbers. Please see http://www.bmj.com/content/340/bmj.c181.long for guidelines on how to de-identify and prepare clinical data for publication. For a list of recommended repositories, please see https://journals.plos.org/plosone/s/recommended-repositories. You also have the option of uploading the data as Supporting Information files, but we would recommend depositing data directly to a data repository if possible.
 
 Please update your Data Availability statement in the submission form accordingly.**

Reviewers' comments:

5. Review Comments to the Author

Please use the space provided to explain your answers to the questions above. You may also include additional comments for the author, including concerns about dual publication, research ethics, or publication ethics. (Please upload your review as an attachment if it exceeds 20,000 characters)

**Reviewer #1**: 
- The authors should more explicitly situate the study within the **psychological and sociological distinctions between adolescence and adulthood. Adolescence is characterized by identity formation, heightened peer orientation, ongoing neural development (particularly in socio-emotional and executive systems), and evolving autonomy from family structures. These features greatly shape how social connectedness is experienced, interpreted, and enacted**. Clarifying these distinctions would significantly strengthen the manuscript’s contribution by demonstrating why findings derived from teenagers cannot simply be extrapolated from adult populations. This clarification should be reflected not only in the literature review but also in the framing of the study’s objectives, the title, and the abstract to foreground the theoretical significance of the work.

Response: Thank you for bringing up these issues. We have amended the manuscript with a section in the background on characteristics of the adolescent period, situating the study within this context of physical and psychological maturation and transition (Rows 38-49). The abstract has been updated to reflect the changes in the manuscript.

- Moreover, adolescence is a critical period for developing durable patterns of social connectedness. Therefore, the manuscript would benefit from **a deeper discussion of how the Swedish socio-cultural context may shape this process. Without such contextualization, the findings risk appearing decontextualized or culturally neutral**.

Response: Thank you for this comment. We have added an elaborated section on socio-cultural context, hopefully contributing to contextualizing the group and the results of the study, please see study setting, rows 123-142.

**- The methodology section requires a more detailed description of the participants and study sites**. The description should explicitly justify how the sample reflects the broader adolescent population in Sweden. If representativeness cannot be ensured, **this limitation** should be acknowledged clearly.

Response: Thank you for bringing this shortcoming to our attention. Further description of the participants and the setting have been added (rows 123-159).

- Furthermore, methodological limitations need to be discussed more explicitly. Potential constraints may include **sampling bias, self-report bias, cultural specificity, limited generalizability, or analytical constraints**. Addressing these issues transparently will enhance the study’s integrity and credibility.

Response: Thank you for this comment. We have added text in the limitations section related to sampling in this section, rows 678-690.

- Finally, the uncertainty–absurdity nexus is omnipresent in adolescents’ lives, with important implications for mental health, the development and erosion of social capital, and patterns of help-seeking. The arguments and insights surrounding this nexus are articulated in a systematic, analytically rigorous manner that preserves both reliability and conceptual generality [1]. Engaging with this framework would help clarify adolescents’ perceptions of the relationship between help-seeking for mental health concerns and emotional connectedness, thereby strengthening the study’s theoretical coherence and enriching the discussion.
[1] Nguyen, M. H. (2026). Uncertainty versus Absurdity: Exploring the Propositions of Wild Wise Weird. https://books.google.com/books?id=Y-3AEQAAQBAJ
I hope these suggestions are helpful.
Cheers,

**Response:** Thanks for this suggestion - we have looked into the suggested references about Uncertainty versus Absurdity by Nguyen (2016). While this offers an interesting theoretical perspective, we feel that adding this perspective may risk drawing attention away from the more central theoretical perspectives in our study, such as theories on help-seeking and developmental theory. Therefore, we have chosen to further elaborate on those theories instead and hope that this has helped to increase the clarity of the study.

Reviewer #2: 

Conceptual Framing. Emotional Connectedness vs. Perceived Meaning

- The manuscript positions “emotional connectedness” as the key construct underpinning help-seeking behaviour. However, the findings suggest that what is most important is not connectedness per se, but adolescents’ interpretation of relational dynamics particularly around trust, confidentiality, predictability, and perceived burden. From a critical realist perspective, it is important to distinguish between the relational structure (closeness/distance), and the adolescent’s interpretation of that structure. Emotional closeness is at times treated as a relational property and at other times as a subjective experience imbued with meaning. Greater conceptual clarity would strengthen the manuscript. Are you analysing relational configurations, or adolescents’ causal beliefs about what makes help-seeking safe or unsafe?

Response: Thank you for this comment. We needed to go back to our manuscript and discuss amongst ourselves before revising the manuscript to be clearer about the concept of emotional connectedness in the results section and discussion. Even though we do not mention the critical realist perspective, we acknowledge the need for clarity. We have tried to be more explicit about emotional connectedness as dynamic relational states mainly based on trust, familiarity, closeness and feeling understood. When participants discussed help-seeking, on the other hand, emotionally close or distant relationships could be perceived as either hindering or facilitating. Thus, the values that these relationships hold can still facilitate or hinder help-seeking depending on contextual circumstances, such as the “cause” of mental health problems, network involvement, social norms or previous experiences etc. We hope that this is now made clearer throughout the manuscript, in the results and in the discussion.

- Relatedly, the manuscript would benefit from stronger engagement with existing help-seeking theory. Without this integration, the contribution risks appearing descriptive rather than theoretically generative.

Response: Thank you for this comment. The background section on help-seeking theory has been revised and we have also added a definition (Rickwood & Thomas, 2012), which helps anchor the study in relevant theory (rows 86-104).


Sample and Transferability

- The study includes 24 adolescents aged 15 from two schools in one Swedish region. While appropriate for qualitative exploration, the manuscript would benefit from clarification regarding mental health status (self-identified distress? clinically assessed?) and discussion of how cultural context may shape relational norms and help-seeking.

Response: Thank you for this comment. We have added information in the methods section, rows 127-142 and 153-159.

Closeness and Burden

- One of the most interesting findings is that emotional closeness can inhibit help-seeking due to fears of burdening others or threatening valued relationships. This resonates strongly with research demonstrating that adolescents’ help-seeking is shaped by perceived consequences for self and others, not merely availability of support. However, this point is not fully theorised in the discussion. Currently, the manuscript stops short of articulating the broader implications of this relational ambivalence.

Response: Thank you for this comment. We agree with your comment and have rewritten parts of the discussion, connecting to theory and clarify the importance of this finding in the implications section. See especially rows 449-468 and 483-488.

Emotional Distance as Facilitative.

- The finding that emotional distance can facilitate help-seeking is interesting. However, the manuscript should clarify if adolescents are distinguishing between emotional intimacy and structural safety. There is a risk that “distance” becomes an umbrella term encompassing multiple mechanisms like anonymity, reduced relational risk, and formal roles. These mechanisms should be disentangled.

Response: Thank you for this comment. We have tried to be clearer about this distinction in our discussion, please see our reasoning on rows 561-573.

Help-Seeking Intention vs Behaviour

- The manuscript refers repeatedly to help-seeking intention. It would be important to clarify whether participants were describing hypothetical scenarios or reflecting on actual past behaviour. This distinction matters, particularly given well established intention behaviour gaps in help-seeking research.
Response: Thank you. We have attempted to clarify in the Data Collection section that the data combined lived experiences with perceptions. There were no inclusion criteria stipulating that the adolescents needed to have had personal lived experience. However, during group conversations, it became evident that some adolescents brought personal knowledge of both mental health problems and the help-seeking process to the table, whereas others did not. In the analysis, we did not differentiate between the two as no records or documentation of “suspected” lived experiences were kept (rows 153-159).

Contribution to Knowledge

- The manuscript’s key contribution appears to be the argument that neither emotional closeness nor distance is inherently facilitative of help-seeking, rather, the relational meaning attached to each determines its influence. This is a nuanced and valuable insight. However, the discussion would benefit from sharper articulation of what is genuinely novel here and how this extends existing literature? It would also be useful to reflect on potential implications for intervention design. For example, interventions that simply encourage “talking to someone close” may be misguided if closeness is perceived as conditional or burdensome. This practical implication deserves stronger emphasis.

Response: Thank you for this comment. We have rewritten parts of the discussion and the conclusion to convey more clearly what we believe to be the major contributions of this study, please see the discussion and the conclusion.

Final statement from authors:

We are thankful for the comments that the reviewers have made. We feel that these revisions have improved our manuscript significantly, and we are grateful for that.

Sincerely/ Mikael, Katrin & Malin
